# Understanding the rheology of nanocontacts

**DOI:** 10.1038/s41467-022-30096-y

**Published:** 2022-05-04

**Authors:** Ali Khosravi, Antoine Lainé, Andrea Vanossi, Jin Wang, Alessandro Siria, Erio Tosatti

**Affiliations:** 1 International School for Advanced Studies (SISSA), I-34136 Trieste, Italy; 2 International Centre for Theoretical Physics, I-34151 Trieste, Italy; 3 CNR-IOM, Consiglio Nazionale delle Ricerche - Istituto Officina dei Materiali, c/o SISSA, 34136 Trieste, Italy; 4 Laboratoire de Physique de lÉcole Normale Supérieure, ENS, Université PSL, CNRS, Sorbonne Université Universitté Paris-Diderot, Sorbonne Paris Cité, UMR CNRS 8550, Paris, France

**Keywords:** Structure of solids and liquids, Nanowires, Computational chemistry, Molecular dynamics

## Abstract

Mechanical stiffness, as opposed to softness, is a fundamental property of solids. Its persistence or rheological evolution in vibrating solid-solid nanocontacts is important in physics, materials science and technology. A puzzling apparent liquefaction under oscillatory strain, totally unexpected at room temperature, was suggested by recent experiments on solid gold nano-junctions. Here we show theoretically that realistically simulated nanocontacts actually remain crystalline even under large oscillatory strains. Tensile and compressive slips, respectively of “necking” and “bellying” types, do take place, but recover reversibly even during fast oscillatory cycles. We also show that, counterintuitively, the residual stress remains tensile after both slips, driving the averaged stiffness from positive to negative, thus superficially mimicking a liquid’s. Unlike a liquid, however, rheological softening occurs by stick-slip, predicting largely frequency independent stiffness with violent noise in stress and conductance, properties compatible with experiments. The baffling large amplitude rheology of gold nanocontacts and its consequences should apply, with different parameters, to many other metals.

## Abstract (web summary)

The rigidity of solid nanocontacts formed when metals touch is apparently lost liquidlike under large mechanical oscillations. As we show theoretically, there is no melting but oscillated nanocontacts undergo a remarkable reversible stick-slip rheology.

## Introduction

The moment two bodies touch, however gently, they do that first through bridging nanoasperities with possible formation of tiny solid junctions, necks, or nanowires, especially in metals. The ability of nanocontacts in realistic room temperature and vibration-rich conditions to transmit, as it were, mechanical rigidity, represents an issue which, even if delicate and important to basic physics, nanoengineering, and even everyday life, has received insufficient attention. Building on historically important work^1^ prototype metallic nanocontacts^2,3^, formed and studied by break junctions, scanning tunneling microscopes (STMs)^4,5^, transmission electron microscopes^6–9^, or cold welding^10^, have been investigated in past decades through conductance, force measurements and simulations, mostly at low temperatures^2,3,11^ or other non-standard conditions^6–9^. Even if thermodynamically metastable against spontaneous thinning and eventual breaking^12,13^, metal nanocontacts can be engineered and mechanically controlled to last long enough to be electrically and magnetically characterized by gate and bias voltages. Their realistic room temperature rheology, however, is an open chapter. Because of short lifetimes associated with nanometric structures and the facile changes they can undergo at ordinary temperature, addressing their non-equilibrium rheological properties requires investigations that go beyond the static and cryogenic conditions of most prototype studies.

In their pioneering experimental exploration of oscillatory stress-strain response of ultra-narrow gold nanojunctions, Comtet et al.^14^ showed that after the initial small strain (0–5%) elastic response, regular plastic yield occurs first, as expected, around 6–7% strain. Recent work by Liu et al.^15^ extended that work to larger cross sections, confirming that with an initially crystalline atomic arrangement inside the nanojunctions, their plastic yield is reasonably attributed to strain-driven slip planes as in macroscopic systems^2,16,17^. However, for larger oscillation amplitudes and atomically thin nanocontacts—with conductances *g* ≈ 3–30*g*_0_ where *g*_0_ = 2*e*^2^/*h*—ref. ^14^ found a further dramatic softening suggestive of apparent liquefaction. The contact effective stiffness with oscillation amplitude above ≈0.15 nm drifted from positive to negative, as would befit a gradually melted nanojunction. How and why large dynamic strains—at room temperature but with mechanical heating totally excluded—could usher in the liquefaction of metal nanoasperities is quite puzzling, requiring a more fundamental understanding of their rheology. Besides the conceptual aspects, if indeed a strongly shaken ordinary metal–metal contact were to consist of myriads of liquefied nanonecks rather than of solid junctions, that would hardly be irrelevant to a host of technological issues.

We theoretically investigate here how, at room temperature, a mechanical perturbation such as a vibration, commonplace in many technological applications, will affect and determine the detailed rheological behavior of contacts including stiffness, dissipation, and yielding under large time-dependent, e.g., oscillatory stresses and strains. Straight theory is ill-equipped to confront this puzzle, owing to both its violently non-equilibrium nature, and its nanometer size scale. It is therefore fortunate that exactly these two features make it directly amenable to non-equilibrium molecular dynamics (NEMD) simulation, which, as we will show, points to an explanation which is different from liquefaction.

## Results

**Fig. 1:**
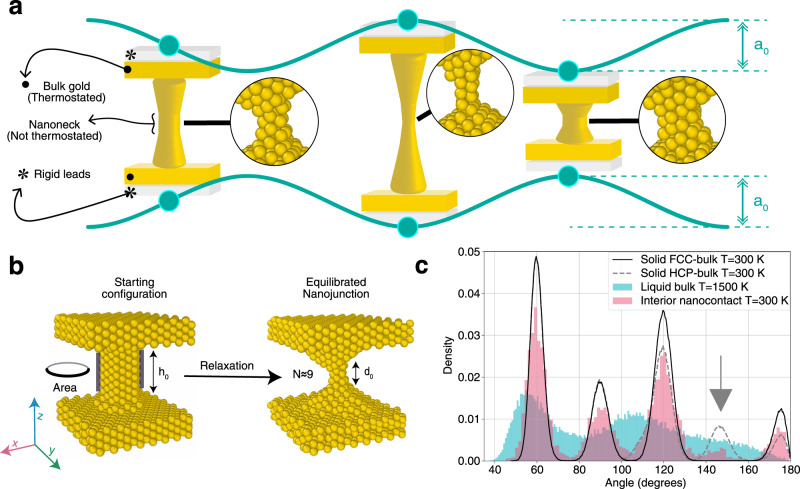
Simulated rheology of an oscillating solid metal nanocontact. **a** Sketch of a gold nanojunction under oscillatory strain, exerted through crystalline leads at room temperature. **b** Snapshots of a nanocontact (*N* ≈ 9 atom cross section) of initial crystalline columnar shape (left) and quasi-equilibrium working shape obtained after extended structural relaxation (right). Note the anvil-like tips formed on both sides of the effective nanojunction's neck reducing its effective length from *h*_0_ to *d*_0_. **c** Three-body angular distribution of inner core atoms of the relaxed junction measured during ≈10 strained cycles at the largest oscillatory amplitude (0.22 nm). The main peaks confirm the survival of fcc structure and coordination of the nanocontact core. The small structure around 147^∘^ (arrow) is a signature of the (111) slips.

**Fig. 2:**
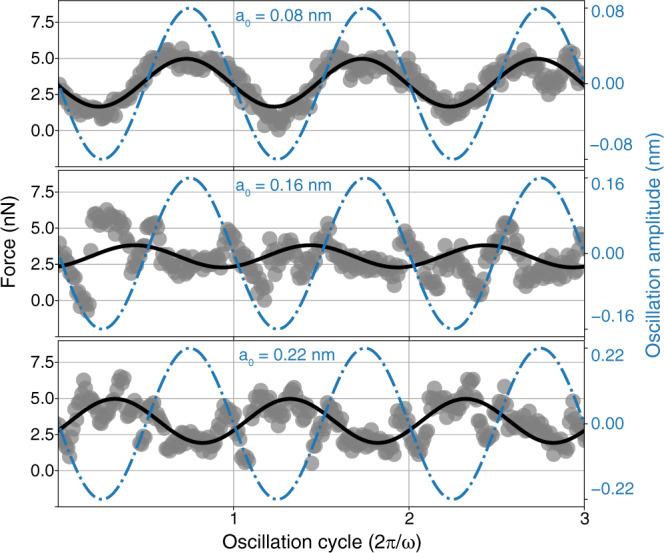
Force time evolution under oscillating strain. Time-dependent force results for the *N* ≈ 9 nanojunction of Fig. 1b with equilibrated shape, initial *h*_0_ ≈ 2.75 nm, relaxed neck length *d*_0_ ≈ 1 nm, average neck cross section area *A* ≈ 0.6 nm^2^, oscillation frequency 50 MHz, *T* = 300 K. Blue dashed-dotted line: imposed lead-lead oscillating amplitude *a*(*t*). Large gray dots: extracted instantaneous force between the leads. Black solid line: sinusoidal fit $F(t)={F}_{0}+(A{\sigma }_{0})\exp (i\omega t+i\phi )$ of the force. The fit parameter *Aσ*_0_ is the resulting force magnitude. Note the change of rheological response with drastic increase of non-sinusoidal noise at and above ${a}_{0}^{* }\approx 0.16$ nm. Note also the prevalence of tensile (positive) force for all strains (see text). The same plot for the largest size of *N* ≈ 26 is shown on Supplementary Fig. 1.

We simulated, suspended between two bulk-like crystalline leads (Fig. 1a) whose distance is oscillated as $h(t)={h}_{0}+{a}_{0}\exp \left(i\omega t\right)$, and read the force time evolution (Fig. 2) to analyse the mechanical response of a family of nanometer radius model gold junctions, under increasing engineering strain amplitude *ϵ*_0_ = *a*_0_/*h*_0_ (conveniently used, although different from true strain *ϵ*_*t*_ = *a*_0_/*d*_0_ where *d*_0_ < *h*_0_ is an effective nanojunction length to be discussed later). All simulations were based on the well-documented and reliable force field of ref. ^18^. We focused on initially bulk-structure columnar junctions bridging between two large solid leads, in our case *h*_0_ ≈ 2.75 nm apart. The initial junction transverse cross section ranged from *N*_*i*_ = 7–40.

The first important step was to establish a realistic nanojunction shape and inner structure. Both are generally unknown experimentally, and expected to evolve in the course of time^12,13^ with shape evolution routes which depend experimentally on temperature and formation protocols^4–6,8,9,14,19^, and theoretically also on the precise form of interatomic interactions^3,18,20–23^. In experimental protocols such as that of ref. ^14^, the unknown nanojunction morphology is, through feedback-actuated preservation of a constant average electronic conductance, periodically stabilized to a roughly constant average cross section area of its narrowest neck. Our simulation protocol started with vigorous initial mechanical oscillations and thermal cycling that transformed the initial idealized column into a modified, relaxed, and reproducible structure that did not further evolve within our subsequent working time. That structure, now consisting of a shorter neck-like nanojunction between spontaneously formed anvil-shaped “tips” survived quasi-stable at room temperature and under further oscillations always comprising no less than 10–20 cycles, with amplitudes up to 0.3 nm. Thus no feedback adjustment was required. The nanojunction retained a well-defined minimal midpoint cross section (Fig. 1b, right) of area $A\approx N\pi {r}_{0}^{2}$, where *r*_0_ ≈ 0.144 nm is the atomic radius and *N* ≈ 4–26 is the atom number inside that cross section—more precisely the number of (110) z-oriented atomic chains crossing that section. In this relaxed configuration, the proper nanojunction length had shrunk from *h*_0_ ≈ 2.75 nm to a smaller value *d*_0_ ≈ 1 nm—now excluding the anvils. The relaxed anvil-nanojunction-anvil shape of the overall contact is not specific to any chosen initial *h*_0_; it just took a shorter relaxation time to realize it for the smallest reasonable lead-lead distance, which we therefore adopted.

**Fig. 3:**
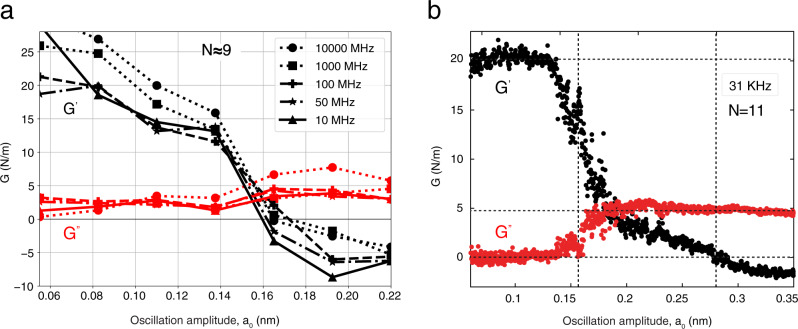
Complex dynamical response function. **a** Effective stiffness $G^{\prime}$ and dissipation *G″* at three decades of frequency 10 MHz–10 GHz for a *N* ≈ 9 atom cross section nanojunction. Similar results are obtained for different sizes and frequencies (see Supplementary Fig. 2 for the largest size of *N* ≈ 26). **b** Experimental data for *N* = 11 at 31 KHz reproduced from ref. ^14^. Note the good overall agreement, parameter-free. For lower frequency data at 1 MHz close to simulation limit, see Supplementary Fig. 8.

The protocol proceeded by submitting the thus relaxed nanojunctions to oscillatory strains with frequencies *ω*/2*π* spanning three decades 10 MHz–10 GHz. Values which, even if much larger, still as we shall see extrapolate naturally down to experimental frequencies such as 31 KHz (see Fig. 3). Preliminary to describing the force results of the simulations, however, the inner atomic structure of the nanojunction and its core is a crucial question to be ascertained. That structure could remain simply fcc crystalline in the first place. It could be glassy—an appealing possibility because glasses are known to liquefy easily under oscillatory strains^24^. Or perhaps the thinnest nanonecks could even possess one of the helically incommensurate coaxial nanotube structures discovered in TEM^6,8^ and theoretically explained^12^ by “magic” string tension minima that arise in the course of spontaneous thinning. Alas, all glassy and helical nanojunction structures which were tried did not survive even for the very first few simulation steps. The latter hypotheses should therefore be discarded.

Direct inspection of both the non-oscillating and oscillating nanojunction interiors in the relaxed structure, actually showed them to be approximately crystalline. The signature is provided by the first-neighbor three-body angular correlation function *ρ*(*θ*) of the interior atoms in the central nanoneck portion, defined by carefully excluding atoms in both the tip anvils and the outer nanojunction surface layer, the latter more mobile than the rest. If the nanojunction interior atoms possessed fcc crystalline coordination, *ρ*(*θ*) should show three peaks at 60^∘^, 90^∘^, and 120^∘^; if liquid or glassy, only 60^∘^ and 120^∘^; if magic, the pattern should be much more complex owing to incommensurability^12^. The result, shown in Fig. 1c shows a clear 60^∘^, 90^∘^, 120^∘^ peak sequence, confirming that the inner core of the simulated nanojunction is and remains close to fcc solid throughout, despite room temperature and violent oscillatory shaking. In addition, the presence in the strongly shaken nanowire of a shoulder around 147^∘^, signals ABC to ABA local sliding of (111) planes during the oscillation, to be discussed below and reminiscent of the yielding patterns proposed in experiments^14,15^.

### Reversible yielding

**Fig. 4:**
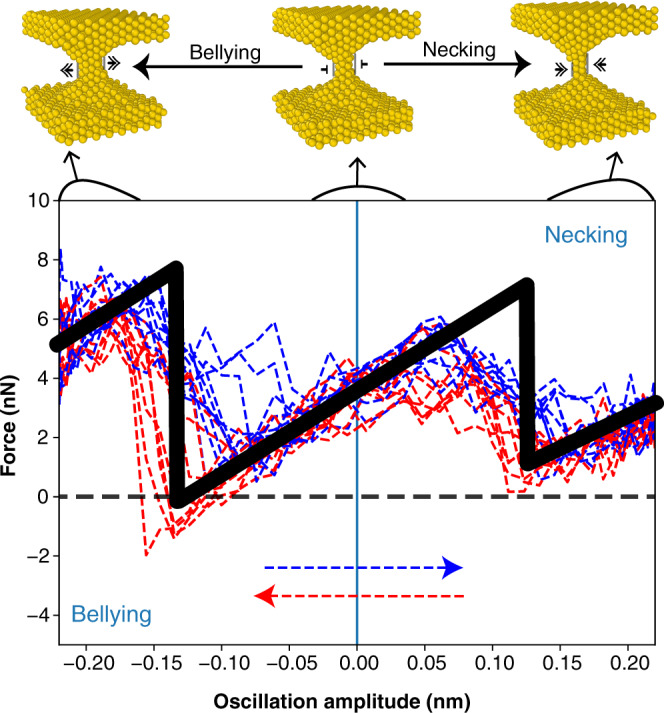
Large amplitude force-strain characteristics. Simulation trajectories in the force-strain plane, for the *N* ≈ 9 nanojunction at 50 MHz. Blue trajectories: half cycle with positive strain time derivative $\frac{d\epsilon }{dt} > 0$; red trajectories: negative strain time derivative $\frac{d\epsilon }{dt} < 0$. Insets are snapshots frames showing the junction at rest (center), after necking (right) and after bellying (left). Black line: schematic adiabatic zig-zag force-strain characteristics. The straight tract near zero is (ignoring fluctuations) the elastic regime. Major yielding slips occurs at necking and bellying, which are the results of forced tensile and compressive strain, respectively. Note the average tension force dominating the whole cycle, surprisingly including the compressive portion, and the large jumps where the force falls near zero at both slips.

The structure evolution under oscillating strain showed up clearly in the simulation geometry, pictured in Fig. 1a, and in Supplementary Fig. 5. As tensile strain amplitude grew, the solid and largely crystalline nanojunction first yielded with a necking local interplane ABC-ABA slip—also causing some thinning. The necking slip took place once the tensile elongation exceeded ≈0.16 nm, which is close to $l/(2\sqrt{2})=0.144$ nm, half of gold’s {110} spacing (the direction of oscillation), where *l* = 0.408 nm is the bulk lattice constant. With some hysteresis, the necking however reversed on the way back, a fast stick-slip-like event made possible on the fly by the subnanometer thickness and by room temperature. In the second half of the cycle, where compression exceeded about the same magnitude in reverse, the nanojunction yielded backward with an inverse necking, which we dub “bellying", for it is accompanied by a noticeable nanojunction thickening. Again with some hysteresis, bellying finally reversed in the last part of the cycle, the junction returning to its initial state. This simulated behavior, whose consequences will also be discussed and pictured later in Fig. 4, exemplifies how reversible plastic deformation kinetics may emerge as a feature in nanocontacts of metals that are, at their working temperature, sufficiently ductile and sufficiently thin. Similar events were earlier reported in the MD simulations of the Au/Ni system by Landman et al.^25^ using EAM potentials. Qualitatively analogous results were also shown by Sutton and Pethica^26^ using a Lennard–Jones pair potential. Even in the bulk crystal, a large (110) uniaxial strain favors hcp relative to fcc, and the local sliding associated with the nanojunction yielding transforms local ABC stacking to ABA (see Supplementary Fig. 5).

### Dynamical force and response function

The structural evolution described underpins the main dynamical output of nanojunctions which we extracted from the oscillatory simulations, that is the instantaneous force *F*(*t*) between the leads. Typical NEMD force time evolution at *ω*/2*π* = 50 MHz are presented in Fig. 2 for increasing oscillation amplitudes *a*_0_, for a nanojunction with relaxed central cross section *N* ≈ 9, chosen as the clearest showcase among all cases studied in the range *N* ≈ 4–26 (see Supplementary Fig. 1 for the largest thickness). At small amplitudes, the mechanical response was essentially elastic—except for some residual fluctuations (possibly connected with the force field’s weak fcc-hcp energy difference^27^)—characterized by a nearly sinusoidal force, and weak phase shift with strain, and thus negligible dissipation. At mechanical yielding, which began rather sharply at a threshold amplitude ${a}_{0}^{* }\approx 0.16$ nm, the force turned noisier (see Supplementary Fig. 3), with sudden jumps associated with the structural interplanar sliding, either necking or bellying, taking place at one point in the nanojunction. From the force, the (conventional) stress magnitude *σ*_0_ was obtained by fitting *F*(*t*) in the form ${F}_{0}+{\sigma }_{0}A\exp (i\omega t+i\phi )$ where *F*_0_ represents a background tensile force (string tension) between the leads at zero strain, *ϕ* the phase shift between the imposed oscillatory strain and the force component of same frequency, and *A* the narrowest cross section area of the relaxed strain-free nanojunction. The force fit (least squares, same frequency as the forcing, amplitude, and phase as parameters) is not an arbitrary choice, but a mandatory protocol required to extract the same linear response function measured experimentally.

The extracted stress magnitude and phase lead to the complex dynamical rigidity $G=G^{\prime} +iG^{\prime\prime} =\left({\sigma }_{0}A/{a}_{0}\right)\exp \left(i\phi \right)$ where the average is taken over a sufficient number of cycles. The real part $G^{\prime}$ is the effective nanojunction stiffness (also called storage modulus), the imaginary part *G″* describes mechanical dissipation (loss modulus). From force data obtained in a vast range of frequencies, nanocontact sizes, and cross sections we extracted the complex dynamical linear response function *G*, shown in Fig. 3a. There is first, as expected, an elastic response at small strains where the stiffness $G^{\prime}$ is positive and large, and dissipation *G″* is, discarding parasitic fluctuations near zero strain (attributable to an excessively small fcc-hcp energy difference, see caption to Supplementary Fig. 2), negligible. As the oscillation amplitudes surpass the yielding magnitude, $G^{\prime}$ drops and eventually turns from positive to negative, corresponding to the force-strain phase reversal that is visible in the raw data of Fig. 2. A corresponding rise of *G″* and a noise increase arose at yielding. The large deviations of force from sinusoidal (detailed in Supplementary Figs. 3 and 4) are responsible for the noise and underscore the inadequacy of linear response description at and after yielding. We nonetheless focus on the linear response because, even if crude, it permits the simplest assessment of kinetics, as well as direct comparison with experiments. The comparison we found with the experimental complex dynamical rigidity is, even if not perfect, definitely convincing. As shown by Fig. 3b^14^ not only the drift of $G^{\prime}$ from positive to negative and the dissipation rise are recovered, but also quantitative values of stiffness and yielding strain are in the right range, without adjustable parameters.

We next investigated the oscillation frequency dependence—the response under variable shear rate is a crucial element providing a clear diagnostic of nanojunction rheology, with different outcomes between a yielding solid and a liquid neck. Ranging from 10 MHz to 10 GHz, the simulated frequency dependence of *G* and its characteristic change of behavior from rigid and elastic to yielding with apparently liquid-like was found in simulations to be essentially nil (see Fig. 3a), suggesting that all important slip phenomena take place very fast. This result is coherent with an energy barrier Δ—estimated across a slip, be it compressive or tensile—larger than *k*_*B*_*T* by at least an order of magnitude, see Supplementary Fig. 7. Similar to friction, the sharp slips lead to a rheological nanojunction behavior close to a sequence of nanoscale stick-slip frictional events, known in turn to give rise to a logarithmically weak or negligible velocity dependence of the friction force^28^. The stick-slip-like rheology of simulated solid nanojunctions and their frequency-independent response over many decades can in addition be extrapolated down to much lower oscillation frequencies—such as those used in experiments, presently out of simulation reach—as follows. There must exist by elementary transition state theory a crossover inverse rate, *ω*_*L*_/2*π* where ${\omega }_{L} \sim {{\Omega }}\exp -{{\Delta }}/{k}_{B}T$ with Ω a mesoscopic attempt frequency scale at which the relevant barrier Δ is thermally overcome during a slip. The basically frequency-independent stick-slip dissipation should persist for oscillation frequency above *ω*_*L*_/2*π*, and eventually cease below, crossing over to so-called thermolubric viscous sliding, linear with frequency^29,30^.

In the *N* ≈ 9 nanocontact the adiabatic energy jumps at slips suggest energy barriers of 0.2–0.4 eV, while Ω ≈ 13 MHz is estimated from real-time force fluctuations close to slips (Supplementary Fig. 7). That predicts a thermolubric crossover frequency estimate *ω*_*L*_/2*π* ~ 600 Hz for this size and temperature. That justifies extrapolation of high-frequency stick-slip behavior to include experimental tuning fork frequencies like 31 KHz^14^, and makes a prediction that might be interesting to verify in future experiments. Our simulations and experiment both show, for all amplitudes above the slip thresholds and up to the largest value, a basically constant *G″* (Fig. 3). At a single frequency, that could, not unreasonably, be interpreted as evidence of viscous response^14^, i.e., *G″* ~ *ηωA*/*h*_0_, where *η* is the viscosity and *A* the area, independent of amplitude. As a function of the primary oscillation frequency, however, our predicted, nearly frequency-independent *G″* is very different from this viscous behavior, at least so long as the frequency does not fall below the thermolubric crossover.

Why there is actually no mechanically induced nanojunction melting in both experiment or simulation is a point already discussed in ref. ^14^, but worth re-examining here. Owing to our large oscillation frequencies, one might suppose that melting could in principle occur the large power *P* provided by the oscillatory strain. In simulation, that power can be evaluated, either as $P=\frac{1}{\tau }\int\nolimits_{0}^{\tau }F(t)\dot{h}(t)dt$ where *τ* = 2*π*/*ω* is the period, or equivalently as the amount of heat absorbed by the thermostat per unit time. For a nanocontact with a cross section *A* ≈ 0.6 nm^2^, oscillating with frequency *ω*/2*π* = 50 MHz and amplitude *a*_0_ = 0.22 nm, one obtains a value of *P* ≈ 1.6 × 10^−11^ W, which, if concentrated on a hypothetical isolated piece of gold of volume *Ad*_0_ ≈ 0.6 nm^3^, would actually melt it in <3 cycles. In a nanojunction, heat is, however, conducted away through the suspending tips to the two leads that are held at *T* = 300 K. Owing to the large electronic thermal conductance, estimated via the Wiedemann-Franz relation (valid also in the ballistic regime, that should actually apply here^31,32^) essentially all heat escapes, leaving only a tiny residue and a temperature increase in the nanojunction that is completely negligible^14^. In our case, simulations actually ignore electronic heat conduction, which in gold is about 95% of the total, thus underestimating the rate of heat escape by a large factor. All the same, the temperature rise in the simulated nanojunction remained negligible, safely below 15 K even at the largest strain amplitudes. The conclusion that the nanojunction does not thermally melt stands therefore absolutely correct. As a further step along that line, considering that a liquid nanoneck could thin down faster than a solid one, we examined the lifetime of *N* ≈ 9 nanojunction when *T* was raised enough to make it locally liquid. Results show (Supplementary Fig. 9) that actual liquid nanonecks at *T* > 500 K should spontaneously evolve and break much faster than any of the experimental feedback and oscillation time scales.

### Intrinsic tensile stress

The agreement of simulated and experimental dynamic response leads us to address with some confidence the question of how a sign change of $G^{\prime}$ at large strains may arise as result of reversible yielding of a solid nanojunction, and why. Reversibility of yielding is in itself not sufficient to account for that, and some additional element must intervene to make the force jumps at necking and bellying large enough, with accumulation of tensile stress, surprisingly even after compressive bellying. That element, theoretically predicted long ago^12,13^ and recently demonstrated in Pt nanowires^19^ is the intrinsically nonzero tensile force—a string tension between the leads—even for a solid nanojunction. In our simulations, tension indeed appeared systematically in all oscillation-free and oscillating nanocontact simulations, as shown by the positive mean force value in Fig. 2. Following even the most careful structural relaxation, which canceled the lead-lead force, tensile stress always resurrected immediately afterward. The bridge-mediated attraction between two bulk-like leads reflects the nanojunction’s intrinsic metastability against thinning and eventual breaking. Atoms in the bridge would, if they could, gain free energy by migrating to the leads. Thinning and breaking, averted in experiments by maintaining a constant average electrical current, have no time to occur in the metastable conditions of a simulation whose duration is much shorter: but the tensile force, transmitted as it were between the leads by the outer nanojunction atoms, is their ubiquitous forerunner. The nonzero average tension force seen in simulations confirm in fact that our rheological results reasonably represent quasi-equilibrium nanojunction conditions. Its value is as it happens, quite easy to predict, ahead of simulations, as follows. Assume a cylindrical nanobridge, solid as well as liquid, with *N*_*t*_ atoms, radius *R*, length *L*, and total Gibbs free energy *H*. The intrinsic lead-lead force is^12^

1 $${F}_{i}=(H-\mu {N}_{t})/L$$

where *μ* is the bulk chemical potential (at *T* = 0, the cohesive energy) per atom. Considering separate surface and bulk contributions to *H*, if the bulk could be considered a true solid, and if the nanocontact possessed well-defined facets, the free energy difference at the numerator could be written as the sum of the surface stress contributions $({\gamma }_{i}+{a}_{i}\frac{d{\gamma }_{i}}{d{a}_{i}})$ where *γ*_*i*_ and *a*_*i*_ are the surface free energy and crystal lattice spacing of the *i*-th facet. If the bulk was liquid instead, that difference would be just the surface free energy *γ*. Neither prescription is perfect for our nanocontacts, that are neither real and faceted true solid portions nor liquid necks. We can nonetheless get a rough estimate by ignoring the difference, not large in gold, between surface free energy and facet-averaged surface stress of the main facets, thus treating it as a liquid. That leads to an intrinsic tensile force, or string tension *F*_*i*_(*R*) = 2*πγR* that is just the total surface free energy 2*πRLγ* divided by length *L*. In our solid simulated junctions this simple force estimate is immediately verified. For example in the *N* ≈ 9 nanojunction of Fig. 2, where *A* ≈ 0.6 nm^2^, whence *R* ≈ 0.44 nm we obtain, inserting gold’s surface energy 0.9 J/m^2^ of the force field^18^, we obtain *F*_*i*_ = 2.5 nN (4.17 nN using instead the experimental surface energy 1.5 J/m^2^) in parameter-free agreement with *F*_0_ ≈ 3.3 ± 1.0 nN of the simulation (the confidence interval is connected with force fluctuations in Supplementary Fig. 3). Similarly, for the *N* ≈ 26 nanojunction, where *A* ≈ 1.7 nm^2^ we predict *F*_*i*_ = 4.2 nN (7.08 nN using the experimental surface energy) and observe *F*_0_ ≈ 5.6 ± 2.2 nN. This large intrinsic lead-lead attraction, capillary-like but present even across a largely solid nanojunction^12,13^, should not be forgotten, when interpreting experiments.

The intrinsic tension and its proportionality to radius *R* represents the second key element to understand the full rheological response. Each time in the cycle the nanojunction undergoes reversible yielding, both necking and bellying, the tensile force and stress undergoes a collapse. Starting from an initially large value which is dictated by the intrinsic tension, down to nearly zero after the slip, whose effect is to cancel essentially the whole force, as demonstrated in Fig. 4.

The effect of intrinsic stress on $G^{\prime}$ can be further demonstrated analytically by e.g., assuming (e.g., for very low-frequency *ω*) a stress-strain behavior *σ*(*ϵ*) that is a hysteresis-free single-valued zig-zag function (see Supplementary Fig. 6)

2 $$\sigma (\epsilon )=k\epsilon -{j}_{n}{{\Theta }}(\epsilon -{\epsilon }_{n})+{j}_{b}{{\Theta }}({\epsilon }_{b}-\epsilon )+{\sigma }_{i}$$

where *k* is the elastic stiffness, *j*_*n*_ (*j*_*b*_) are the necking (bellying) stress jumps, Θ is the Heaviside function, and *σ*_*i*_ = *F*_*i*_/*A* is the intrinsic stress at zero strain. The effective stiffness $G^{\prime}$ (strictly real in this case) is the ratio of the Fourier transforms *σ*(*ω*) and *ϵ*(*ω*)

3 $$G^{\prime} =	 \,\frac{2i}{{\epsilon }_{0}}\frac{\omega }{2\pi }\int\nolimits_{-\pi /\omega }^{\pi /\omega }\sigma [{\epsilon }_{0}\exp (i\omega t)]\exp (-i\omega t)dt\\ =	 \,k-\frac{2}{\pi {\epsilon }_{0}}[{j}_{n}\sqrt{1-{({\epsilon }_{n}/{\epsilon }_{0})}^{2}}+{j}_{b}\sqrt{1-{({\epsilon }_{b}/{\epsilon }_{0})}^{2}}].$$

The last two terms show how the stress drops *j*_*n*_ and *j*_*b*_—that are large because of the large intrinsic stress—effectively act to reverse the sign of $G^{\prime}$ when *ϵ*_0_ exceeds the yielding thresholds *ϵ*_*n*_ and ∣*ϵ*_*b*_∣ (see Supplementary Fig. 6).

## Discussion

Summing up, we have found that the large-strain rheology of a ductile metal nanojunction, here exemplified by room temperature gold, is dominated by two elements: reversible yielding, and a large size-dependent intrinsic tensile stress, both already apparent in the raw simulation data of Fig. 2. The reversible yield slips occur in a basically crystalline structure, as opposed to a conjectured strain-driven liquefaction. The size-dependent tensile stress, predicted to exist in a metal nanojunction in quasi-equilibrium at finite temperature, even if close to a solid inside, causes the stress jumps to be large. The two elements concur to change the sign of the effective stiffness $G^{\prime}$ from positive to negative, a reversal close to that seen in experiments. The dissipative response *G″* at large oscillation amplitudes is predicted to behave as stick-slip friction, that is with weak or negligible frequency dependence. If the experimental tuning fork frequencies could hypothetically be extended to cover higher values they should directly exclude, we believe, the dramatic linear growth expected for a liquid. If on the other hand the oscillation frequency could hypothetically be lowered enough to reach *ω*_*L*_, or alternatively if *ω*_*L*_ could be raised enough by increasing temperature, then stick-slip should be found to cross over to thermolubric sliding. A cartoon of the full response in the limit of vanishing *ω*, for example, is that portrayed in the zig-zag model of Supplementary Fig. 6, where $G^{\prime}$ still switches from positive to negative, whereas dissipation vanishes, *G″* = 0. A predicted additional consequence of the reversible stick-slip rheology is a large anharmonicity and associated force noise, also be accompanied by conductance noise. Clearly reported in experiments^14^, such a large noise cannot be easily rationalized for a liquid neck.

Investigated here in the specific instance of gold, a very ductile metal at room temperature, the conditions under which a vibration-induced nanojunction softening might occur in different metal contacts of technological importance should be pursued in future studies. Fully developed intrinsic tension in generic nanocontacts might be hampered by slower kinetics in either less ductile metals or ones with larger slip energy barriers (thus showing up only at higher temperatures), where nonetheless the same conclusions about the non-equilibrium dynamics of nano and atomic contacts should apply. The mechanical and rheological behavior under oscillatory strain induced by vibrations of the specific case of gold junctions, expected to be technologically important in electrical contacts^33,34^—should be of high interest for gold-plated connectors and switches in industrial applications^35,36^, for spacecrafts evolving in microgravity^37^ and a multitude of other strongly vibrating contacts. Potential applications beyond the case of gold could play a role in adhesion and friction of vibrating systems, where negative stiffness could alter the mechanical stability, the friction, and the electronic performance of more general metal nanocontacts. Our findings may have major impact in emerging industrial applications such a direct printing of metallic wires at the nanoscale. Indeed the increased stability due to shear induced negative stiffness can prevent mechanical instability of metallic wire and dewetting of surface at the nanoscale providing a novel class of electronic devices.

## Methods

The sub-microsecond simulations were carried out using the Large-scale Atomic/Molecular Massively Parallel Simulator (LAMMPS) open-source code^38^. Following previous work by Park and Zimmermann^39^, and many other studies, we adopted the realistic embedded-atom method (EAM) gold force field by Foiles et al.^18^. The simulation setup consisted of 2042–2516 particles (*N* ≈ 9–26), with periodic boundary conditions (PBC) in (x,y) plane and free boundary conditions along z axis (the strain axis). The two upper and lower gold leads consist of two fcc rigidly stacked (110) lattice planes. Four thermostating planes consisting of 576 atoms are attached to the leads at each side, joining together with an initial 2.75-nm long column with various thicknesses. The setup is thermally and mechanically relaxed, as shown Fig. 1b, finally evolving into a shorter nanojunction subtended between two spontaneously formed anvil-shaped tips. An oscillatory z-displacement is added to the lead-lead distance, causing the nanojunction to undergo a tensile and compressive deformation. Non-equilibrium molecular dynamics (NEMD) simulation is carried out in the framework of Langevin approach at room temperature. In order to minimize the possible influence of this simulating procedure, the thermostat is applied just to the mobile atoms outside the physically relevant region defined by *h*_0_. Besides, we checked that, within a significant range of values of the Langevin damping parameter, rheological response of the system is reasonably independent of its specific choice. The vertical force between two leads are read directly from simulations, the phase shift between force signal and the imposed oscillation is the concern of analysis in this work.

## Supplementary information

Supplementary_Information

## Data Availability

The data that support the findings of this study are available from the corresponding author upon request.
