# Supplementary material for: Understanding the rheology of nanocontacts
Source: Nat Commun. 2022 May 4;13:2428. doi: 10.1038/s41467-022-30096-y (PMC9068906; doi:10.1038/s41467-022-30096-y)
Supplement: Supplementary file 1 — Supplementary_Information [file 41467_2022_30096_MOESM1_ESM.pdf]

Supplementary Information
Understanding the Rheology of Nanocontacts

Ali Khosravi et al.

Supplementary Notes 1: Robustness of results

Simulations have been performed on nanojunction of varying lateral size in order to investigate the validity of the proposed approach. Almost identical rheological response versus increasing strain oscillations is observed also in nanocontacts with larger mid-junction thickness (here, e.g., $N \approx 26$). The evolution of the force-strain phase differences with oscillating amplitude is shown in Supplementary Fig. 1.

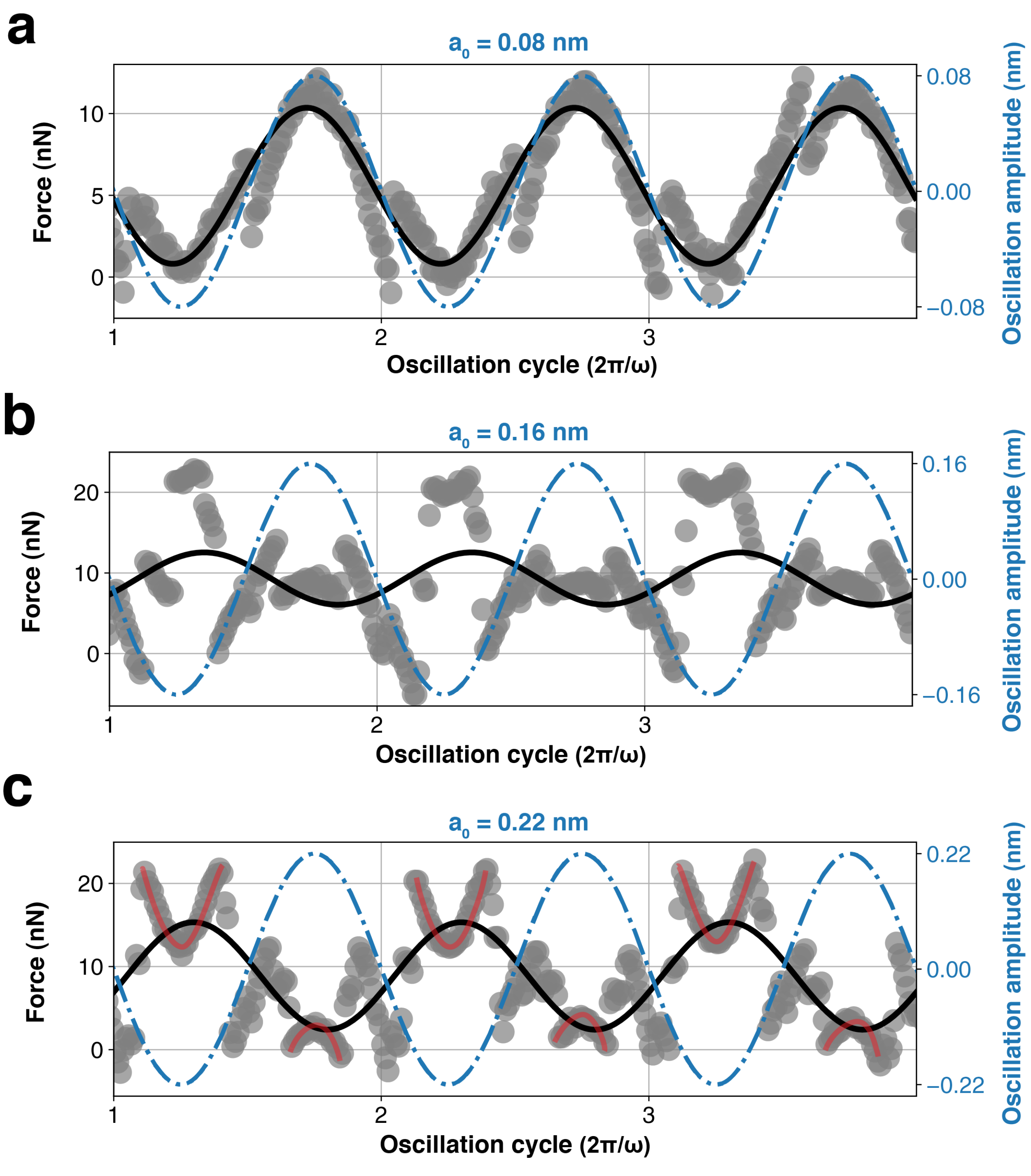

Supplementary Figure 1: **Force time evolution of a thicker nanojunction under oscillating strain** Time dependent force results (oscillation frequency 50 MHz , T=300 K) for a thicker ($N \approx 26$) nanojunction, with initial $h_0 \approx 2.75$ nm, relaxed neck length $d_0 \approx 1$ nm, and average neck cross section area $A \approx 1.7$ nm^2. Blue dashed-dotted line: imposed lead-lead oscillating distance $a(t)$. Large grey dots: extracted instantaneous force between the leads. Black solid line: sinusoidal fit $F(t) = F_0 + (A\sigma_0)\exp(i\omega t + i\phi))$ of the force. Note how avalanche-like jumps (in red), occurring at the maxima of the driving strain, gradually inverts the sinusoidal fit phase, moving from small **(a)** towards large **(c)** imposed amplitudes. Once again, the prevalence of tensile (positive) force shows up for all strains.

In connection with what is already shown in main text for a thinner nanocontact, Supplementary Fig. 2 displays the $N \approx 26$ nanojunction force-strain characteristics and the corresponding complex dynamical linear response function, in basic agreement with smaller sizes.

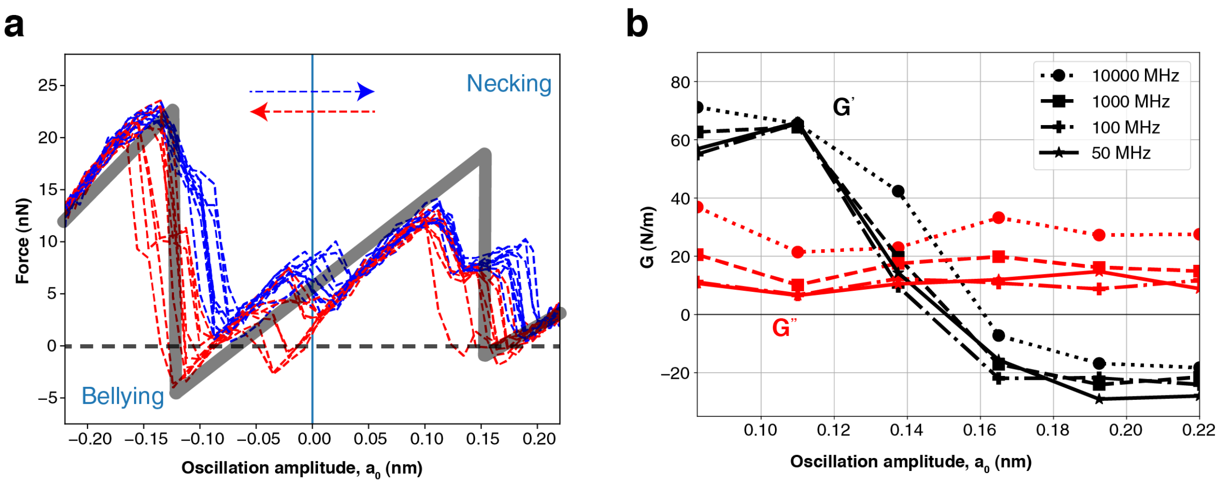

Supplementary Figure 2: **Rheology of the thicker ($N \approx 26$) nanojunction at 50 MHz strain rate**. **(a)** Large amplitude simulation trajectories of a thick nanojunction in the force-strain plane. Blue trajectories: half cycle with positive strain time derivative $\frac{d\epsilon}{dt} > 0$; red trajectories: negative strain time derivative $\frac{d\epsilon}{dt} < 0$. Black line: schematic (adiabatic) zig-zag force-strain characteristics. At small amplitudes, the straight tract represents an essentially elastic regime, except for some residual fluctuations possibly connected with the force field's small (1.1 $mev/atom$) fcc-hcp energy difference [1]. Major yielding instabilities occurs at necking and bellying ($a_0^* \approx 0.16$ nm), which are the results of forced tensile and compressive strain, respectively. As observed for the thinner nanocontact, the average tension force dominates the whole cycle, including the compressive portion, causing the large jumps where the force falls near zero at the yielding strains. **(b)** Effective stiffness G' and dissipation G'' of the complex dynamical linear response function G. Note how the overall behaviour is frequency independent, one of the evidence for non-liquidity of the junction.

Supplementary Notes 2: Force fluctuations, nonlinear noise spectrum

The simulated force between the two oscillating leads fluctuates very violently after yielding, and especially at large strain frequencies, where the instantaneous force deviates from the sinusoidal linear response fit.

At all frequencies, and for both thin $N \approx 9$ and thick $N \approx 26$ nanojunctions (see Supplementary Fig. 3), fluctuations tend to rapidly increase toward yielding ($a_0 \approx 0.165\ nm$), signaling the gross inadequacy of linear response description beyond the elastic regime.

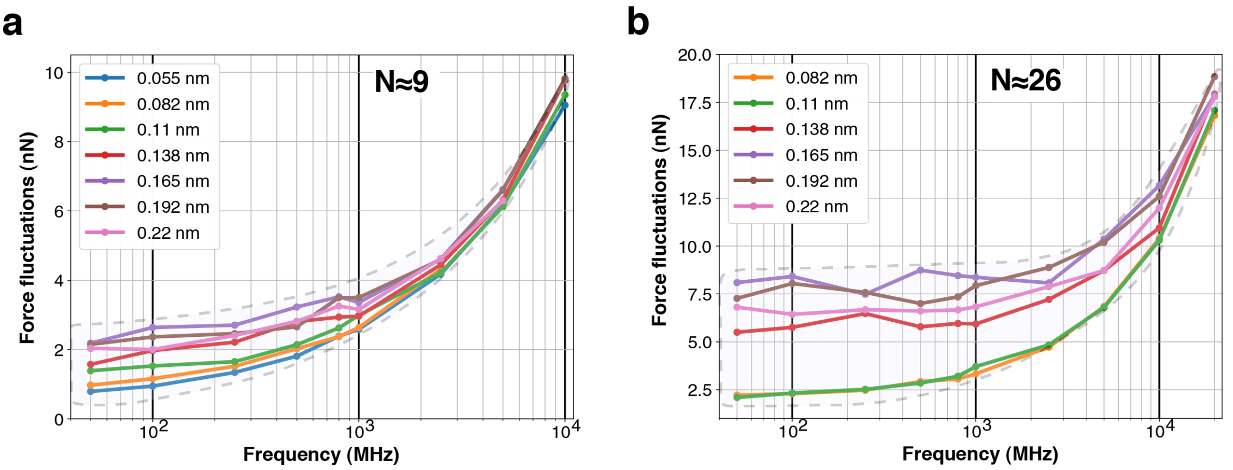

Supplementary Figure 3: **Force fluctuations frequency dependence, extracted for different strain oscillation amplitudes** for $N \approx 9$ **(a)** and $N \approx 26$ **(b)** nanojunctions, respectively. For each parameters set, data is extracted over many oscillation cycles, considering half width at half maximum of Gaussian distribution of the recorded force signal deviating from its sinusoidal linear response fit.

In addition, we Fourier analyzed the lead-lead force

$$F(t) = \frac{a_0}{2} + \sum_{n=1}^{\infty} a_n \cos(n\omega_0 t) + b_n \sin(n\omega_0 t) \tag{1}$$

to obtain the noise spectrum in the two relevant regimes: a) small strain amplitude, where without slips the nanojunction is basically elastic, with positive stiffness and b) large strain amplitude, where the nanojunction deforms with reversible slips and stiffness is negative. As shown in Supplementary Fig. 4 for the nanojunction of $N \approx 9$ the noise spectrum broadens dramatically in stick-slip regime (b), with large higher harmonic contents at $\omega = n\omega_0$ besides the fundamental at $\omega = \omega_0$. The peak n values depend on the oscillation amplitude, reflecting the changeable position of slips within the cycle.

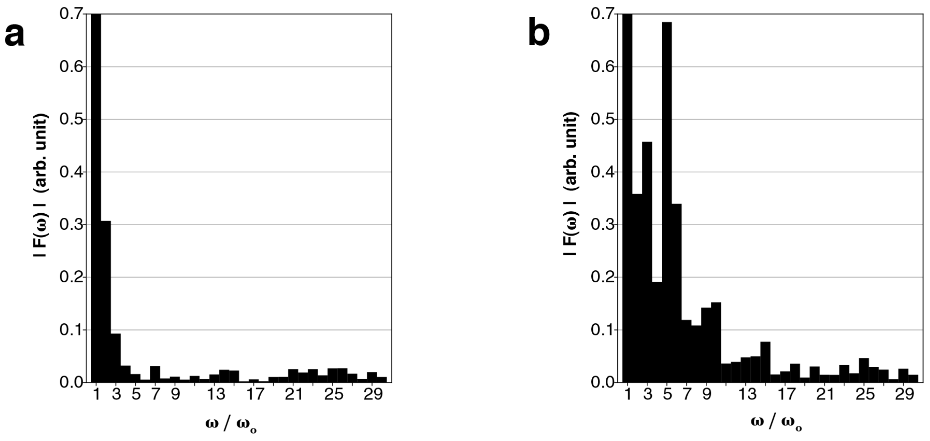

Supplementary Figure 4: **Noise spectrum for $N \approx 9$** at small amplitude $a_0 = 0.08$ nm **(a)**, frequency 50 MHz and large amplitude $a_0 = 0.22$ nm **(b)**. The frequency scale shows $n = \omega/\omega_0$. The $|F(\omega)|$ histograms show $\sqrt{a_n^2 + b_n^2}$, normalized so that $F(\omega_0)$ =1. Note the broad-spectrum noise generated by stick-slip at large amplitudes.

Supplementary Notes 3: Nanocontact structural slips

Here, we show how the force drops, responsible for the zig-zag character in the force-strain trajectories (see Supplementary Fig. 2), are caused by local structural slips. To explore this feature, we perform an oscillatory athermal ($T = 0$) simulation with strain amplitude $0.22nm$, where layer ordering is easily detectable in the $N \approx 9$ nanowire as shown in Supplementary Fig. 5a.

To verify the structural consequences of avalanche-like instabilities at each strain cycle, we successively determine the angular distribution, now at room temperature, for the $N \approx 26$ nanocontact – central atoms only, excluding surface ones – at 50MHz. Supplementary Fig. 5b reports the angular distribution of the nanojunction structure right when it experiences the largest ($0.22nm$) tensile and compressive strain within the cycle. The shoulder around 147° signals precisely ABC to ABA local sliding of (111) planes after necking. That same feature is missing after the bellying avalanche, where a final fcc structure is restored under compression, a regime where hcp structure is disfavored.

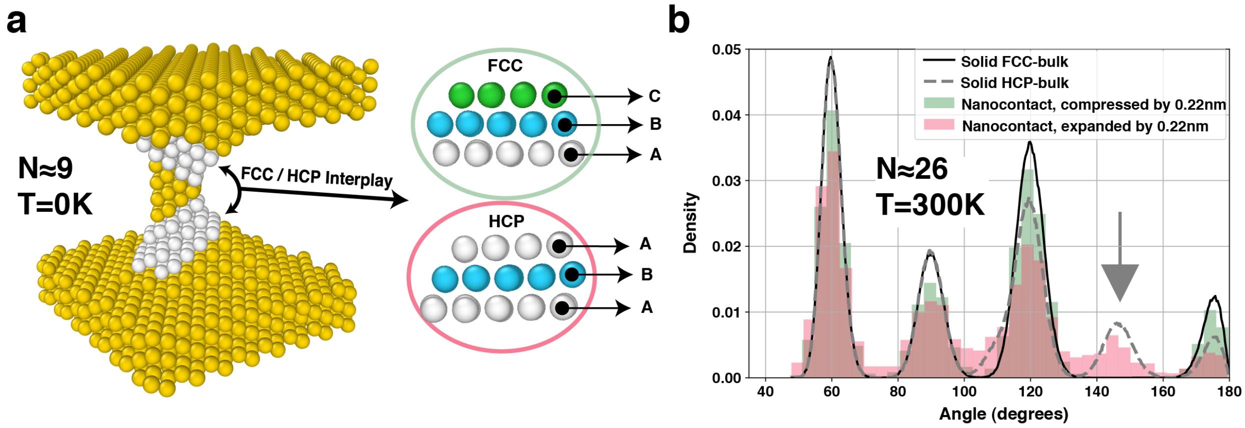

Supplementary Figure 5: **Nanocontact structural and interplanar sliding features**. **(a)** Local structural slip during a $0.22nm$ strain oscillation for the $N \approx 9$ nanocontact, at zero temperature. **(b)** Angular distribution at maximum compressive/tensile strain (0.22nm) of the thicker $N \approx 26$ nanojunction at 300K. The dark arrow highlights the appearance of a shoulder around 147° signaling the ABC to ABA interplanar local sliding after necking. Simulated frequency is 50MHz.

Supplementary Notes 4: Zig-zag model

The simple toy model introduced in the main text and characterized by

$$\begin{aligned}\sigma(\epsilon) &= k\epsilon - j_n\Theta(\epsilon(t)-\epsilon_n) + j_b\Theta(\epsilon_b - \epsilon(t)) + \sigma_i \\ \epsilon(t) &= \epsilon_0 \sin\omega t\end{aligned} \tag{2}$$

where k is the elastic stiffness, and j_n (j_b) are the necking (bellying) jumps at yield strain ϵ_n (ϵ_b), and σ_i is the intrinsic stress at zero strain, can help us to understand the role of vertical jumps in determining the values of G'. As already mentioned, the effective stiffness can be calculated as ratio of the Fourier transforms

$$\begin{aligned}\epsilon(\omega) &= \frac{\omega}{2\pi}\int_{-\pi/\omega}^{\pi/\omega} \epsilon(t)\exp(-i\omega t)dt = \frac{\epsilon_0}{2i} \\ \sigma(\omega) &= \frac{\omega}{2\pi}\int_{-\pi/\omega}^{\pi/\omega} \sigma(\epsilon(t))\exp(-i\omega t)dt \\ &= \frac{k\epsilon_0}{2i} - \frac{j_n}{\pi i}\sqrt{1-(\epsilon_n/\epsilon_0)^2} - \frac{j_b}{\pi i}\sqrt{1-(\epsilon_b/\epsilon_0)^2} + 0\end{aligned} \tag{3}$$

leading to the formula:

$$G' = \left.\frac{\sigma(\omega)}{\epsilon(\omega)}\right|_{\omega=\omega_0} = k - \frac{2}{\pi\epsilon_0}\left[j_n\sqrt{1-(\epsilon_n/\epsilon_0)^2} + j_b\sqrt{1-(\epsilon_b/\epsilon_0)^2}\right] \tag{4}$$

This is the exact equivalent of least square fitting protocol we employed to extract G' from the MD simulations.

For several values of j_n and j_b – renamed simply j here –, Supplementary Fig. 6 shows the zig-zag response of this toy model, and the corresponding effective stiffness G' calculated from Eq. (4). The stress jump amplitude j plays a crucial role in determining G' sign reversal. When the applied strain is still within the elastic regime, the overall slope in Supplementary Fig. 6a remains positive; at the yield slips however, both necking and bellying, the stress drops to zero, turning negative the overall slope, and thus G'.

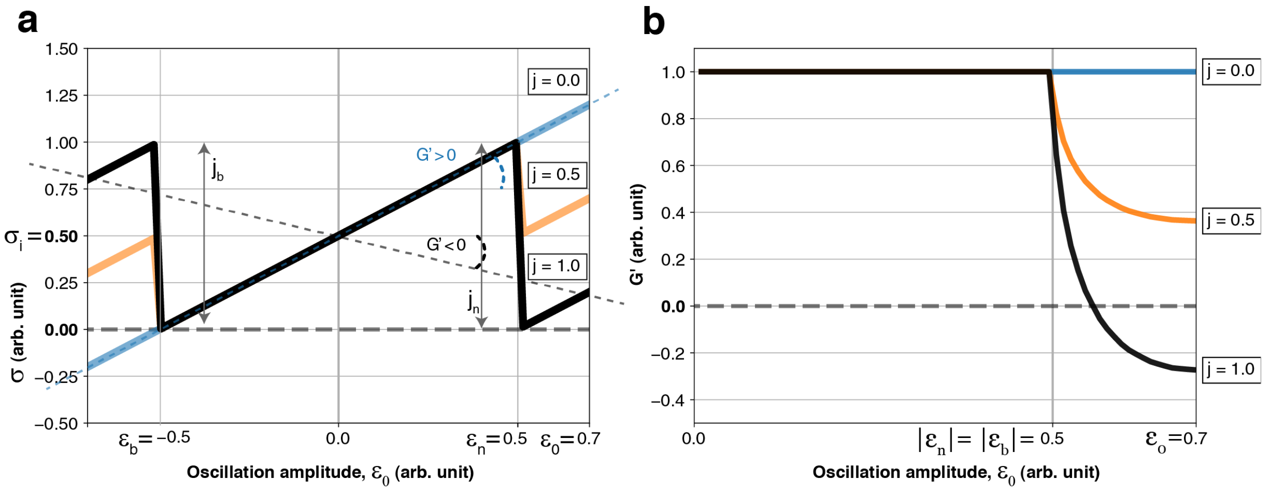

Supplementary Figure 6: **Zig-zag stress-strain model for parameters: $j = j_n = j_b$, $\epsilon_n = |\epsilon_b| = 0.5$, $\epsilon_0 = 0.7$, $\sigma_i = 0.5$, and $k = 1$.** **(a)** Model stress-amplitude characteristics, mimicking the avalanche-like slips in the simulated nanojunctions. The overall slope highlighted by dashed lines represents a rough approximation of G'. **(b)** G' calculated using equation Eq. (4). Note how G' turns negative for $j = 1$, the value making the stress jumps touch zero, as in simulations. The model demonstrates how a large average tensile stress, imposes also a large value of stress jumps, j –as the result of touching zero– necessarily for G' to turn negative.

Supplementary Notes 5: Energy barriers and attempt frequency

The regime adopted by each strain-induced slip of the nanojunction may be rationalized by standard transition state theory, where the inverse rate

$$2\pi\tau^{-1} = \omega_L = \Omega \exp -\Delta/k_B T \quad (5)$$

is controlled by an energy barrier Δ and an attempt frequency Ω. While both quantities depend on specific nanojunction and its conditions, we obtained an order of magnitude of the barriers from the total energy evolution of the athermal nanocontact of Supplementary Fig. 5a, and extract a rough value between 0.2 and 0.4 ev of the energy jumps, proportional to the barriers (possibly slightly underestimated by the force field), associated with single slips (Supplementary Fig. 7a). We also ran a 300K non-oscillatory simulation of the nanocontact ($N \approx 9$), pre-strained by -0.12 nm, a regime where unbellying slip occur erratically and attempt oscillations are more conspicuous. The analysis of Supplementary Fig. 7b-c yields an estimated $\Omega \sim 13.3$ MHz for the attempt frequency of the N=9 nanocontact. Assuming qualitatively therefore, a barrier of 10 $k_B T$ and an attempt frequency Ω as found we obtain a crude crossover frequency estimate $\omega_L \sim 600$ Hz.

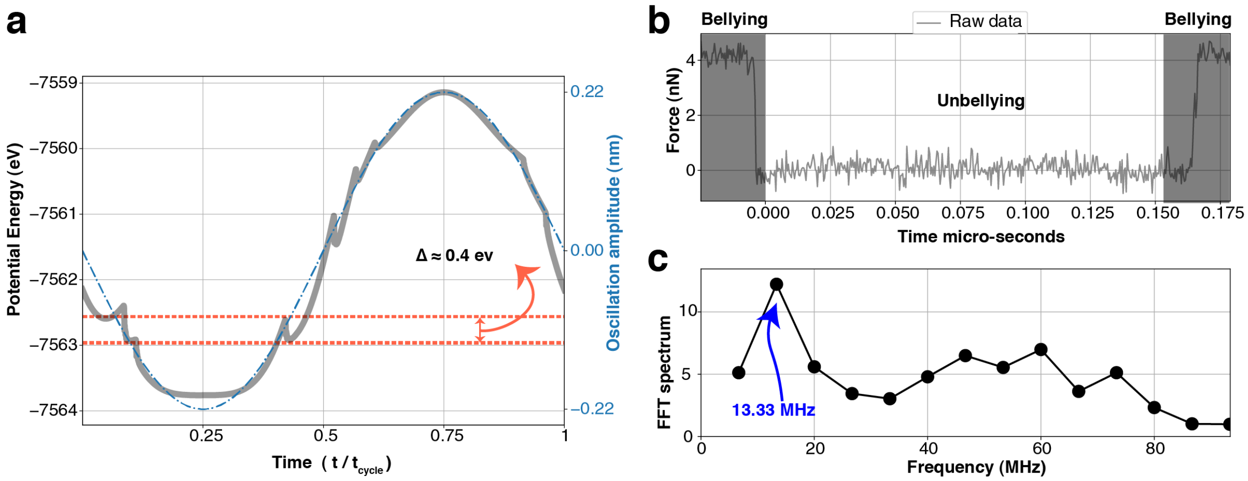

Supplementary Figure 7: **Analysis of the force noise and energy jumps near and following an "unbellying" slip**. **(a)** Evolution of total energy during an adiabatic strain oscillation at T=0 in the $N = 9$ junction. Two orange dashed lines show a typical energy barrier of a single slip, e.g., bellying. The jumps provide an order of magnitude of the slip energy barriers. **(b)** Raw force signal from simulation at T=300K **(c)** Fourier spectrum of force, showing weak attempt frequency oscillations centered at $\omega = 13.3$ MHz

Supplementary Notes 6: Heat conductance

The heat conductivity of a metal such as gold is largely electronic. Thus the mechanical heat generated inside the junction by oscillatory strain is basically conducted away by electrons at the Fermi surface. The heat conductance in the bulk metal is related to electrical conductance through the Wiedemann-Franz relation. For our nanojunctions, however, since their typical length is smaller than the electron's mean-free-path –37.7 nm for gold[2]–, electrical conductance is ballistic, and not diffusive as in bulk. It has been conclusively shown that the Wiedemann-Franz relation holds also in ballistic nanochannels[3; 4], which for the nanojunction with cross section $N \approx 9$ at 300K for instance, gives:

$$\kappa_{ballistic} = N \times \frac{\pi^2 k_B^2 T}{3h} \approx 2.56 \times 10^{-9}\ WK^{-1} \tag{6}$$

Using this value, we can estimate what would be the increase in temperature in an ideal nanojunction, in presence of the ballistic electronic heat conductance. With a mechanical Joule power of 2 ev/cycle $\sim 1.6 \times 10^{-11}$ W in the case of $N \approx 9$ while oscillating with amplitude of 0.22 nm at frequency of 50 MHz, the expected temperature increase would be

$$\Delta T = \frac{1.6 \times 10^{-11}}{2.56 \times 10^{-9}} \approx 0.006\ K \tag{7}$$

In our MD simulations the electronic conductance, responsible for about 95%[4] of total heat conductance in a real gold nanojunction, is of course absent. Moreover, we only thermostat the two thin solid lead slabs supporting the whole contact of length h_0 (see Fig. 1). Unsurprisingly we observe a higher simulated temperature rise of the interior nanocontact than that estimated above, but still below 15 K, thus unable to cause melting, and altogether irrelevant.

Supplementary Notes 7: Low frequency simulations

For a qualitative check and validation of our results, we lower the frequency down to 10 MHz and 1 MHz, performing in the latter one oscillation cycle only. Less accurate as that result is, the response function extracted and shown in Supplementary Fig. 8 shows that the overall conclusions drawn in text remain valid. Specifically, the reversible yield slips, the sign change of G' with frequency independent G" at large amplitudes are confirmed.

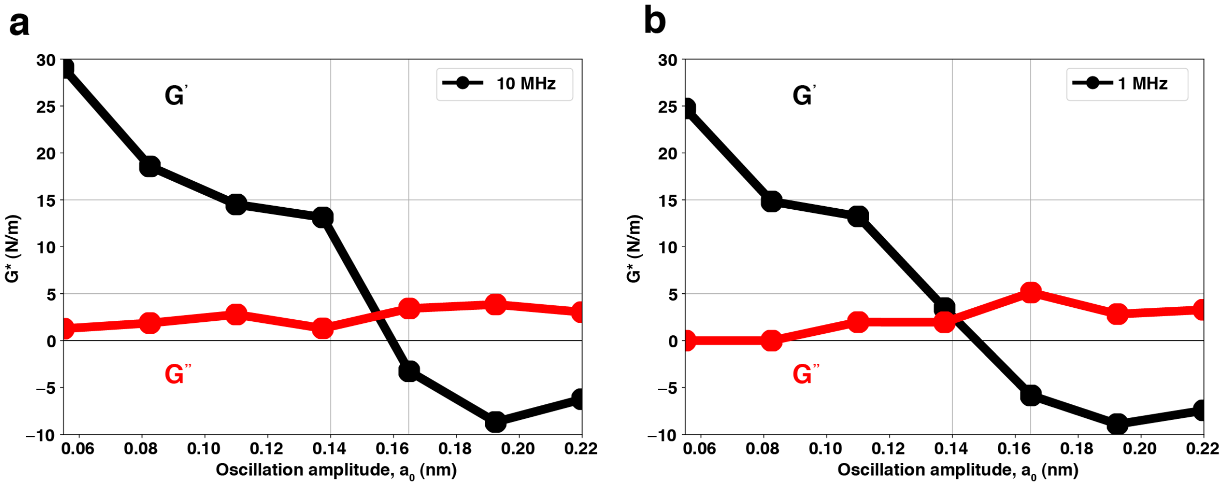

Supplementary Figure 8: **Complex dynamical linear response function G, for nanocontact $N \approx 9$. (a)** 10 cycles, 10 MHz **(b)** 1 cycle, 1 MHz

Supplementary Notes 8: High temperature nanocontact lifetime

The experimental time scale to control the contact size is many orders of magnitude larger than the life time of a heated nanocontact. We conclude experimental nanocontacts are always solid.

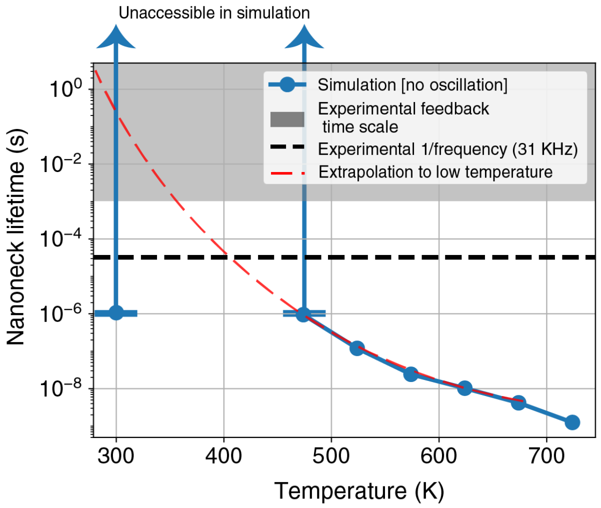

Supplementary Figure 9: **Nanocontact lifetime of a $N \approx 9$ nanocontact obtained as a function of temperature without vertical oscillation, simulation duration is less than 1μ sec.** Black dashed line and grey band are the time period and experimental feedback time range used in Ref. [5]. Red dashed line: lifetime extrapolation at low temperatures. Liquid nanonecks at $T > 500$ K break extremely fast, while already at 400 K the lifetime would be shorter than the experimental feedback time.

Supplementary References

[1] H. W. Sheng, M. J. Kramer, A. Cadien, T. Fujita, and M. W. Chen. Highly optimized embedded-atom-method potentials for fourteen fcc metals. *Phys. Rev. B*, 83:134118, 2011.

[2] Daniel Gall. Electron mean free path in elemental metals. *Journal of Applied Physics*, 119(8):085101, 2016.

[3] Nico Mosso, Ute Drechsler, Fabian Menges, Peter Nirmalraj, Siegfried Karg, Heike Riel, and Bernd Gotsmann. Heat transport through atomic contacts. *Nature Nanotechnology*, 12:430–433, 2017.

[4] Longji Cui, Wonho Jeong, Sunghoon Hur, Manuel Matt, Jan C. Klöckner, Fabian Pauly, Peter Nielaba, Juan Carlos Cuevas, Edgar Meyhofer, and Pramod Reddy. Quantized thermal transport in single-atom junctions. *Science*, 355(6330):1192–1195, 2017.

[5] Jean Comtet, Antoine Lainé, Antoine Niguès, Lydéric Bocquet, and Alessandro Siria. Atomic rheology of gold nanojunctions. *Nature*, 569(7756):393–397, 2019.
